# Erythropoietin protection effects against ischemia-reperfusion injuries during coronary artery bypass graft surgery: a randomized, double blinded, placebo control study

**DOI:** 10.1186/1749-8090-8-S1-O209

**Published:** 2013-09-11

**Authors:** SH Ziabakhsh Tabary, F Mokhtari-Esbui

**Affiliations:** 1 Mazandaran University of Medical Sciences, Mazandaran Heart Center, Sari, Iran

## Background

Although the rapid reperfusion by coronary artery bypass graft (CABG) surgery has significant success and it can cause decrease in mortality and morbidity rate, but this reperfusion paradoxically can cause ionic and metabolic damage that lead to myocardial damage and myocytes death. We examined whether perioperative exogenous erythropoietin (EPO) can reduce myocardial damage by reducing troponin I and creatine kinase (CKMB) level.

## Methods

43 patients randomly divided into two groups. Patients in erythropoietin group were treated by common medical therapies and CABG plus 700 IU/kg erythropoietin (PD Poietin, puyeshdaroo, Iran), intravenously infusion, exactly 5 min after termination of cross clamp: at the start of reperfusion and patients in control group were treated by common medical therapies and CABG surgery plus 10cc normal saline as placebo. CKMB and Troponin I, were measured before and 8 hours after surgery. Echocardiography was performed 4days after surgery in all patients.

## Results

No differences were detected between EPO and control group in change of troponin from before to after surgery (1.41±2.09 vs. 1.79±1.42 with P= 0.49). There were no significant differences between change of CKMB from before to after surgery in EPO and control group (20.59±17.51 vs. 11.57±14.46 with P=0.07). Also Wall motion score index (WMSI) level, 4 days after operation was nearly the same in erythropoietin and control groups. (1.08 ±0.09 vs. 1.07 ±0.10 with P= 0.83).

## Conclusions

In our study no inversely correlation were detected between infusion of erythropoietin and change of Troponin I and CKMB after CABG surgery. Erythropoietin had no effects on reduction of remodeling and stunning of ventricular septum or improving ventricular function in early postoperative period.

